# Rational case management of malaria with a rapid diagnostic test, Paracheck Pf®, in antenatal health care in Bangui, Central African Republic

**DOI:** 10.1186/1471-2458-12-482

**Published:** 2012-06-26

**Authors:** Alexandre Manirakiza, Eugène Serdouma, Luc Salva Heredeïbona, Djibrine Djalle, Nestor Madji, Methode Moyen, Georges Soula, Alain Le Faou, Jean Delmont

**Affiliations:** 1 Institut Pasteur de Bangui, Bangui, PO Box 923, Central African Republic; 2 Centre de Formation et de Recherche en Médecine et Santé Tropicales, Faculté de Médecine Nord, 13015, Marseille, France; 3 Reproductive Health, Ministry of Public Health, Population and AIDS Control, PO Box 883, Bangui, Central African Republic; 4 Faculty of Health Sciences, University of Bangui, PO Box 1383, Bangui, Central African Republic; 5 Ministry of Public Health, Population and AIDS Control, Castors Health Centre, Bangui, Central African Republic; 6 Malaria Programme Division, Ministry of Public Health, Population and AIDS Control, PO Box 883, Bangui, Central African Republic; 7 Hôpital de Brabois Adultes, CHU de Nancy, 54511, Vandoeuvre-lès-Nancy Cedex, France

**Keywords:** Rapid diagnostic test, Malaria, Pregnancy

## Abstract

**Background:**

Both treatment and prevention strategies are recommended by the World Health Organization for the control of malaria during pregnancy in tropical areas. The aim of this study was to assess use of a rapid diagnostic test for prompt management of malaria in pregnancy in Bangui, Central African Republic.

**Methods:**

A cohort of 76 pregnant women was screened systematically for malaria with Paracheck_Pf_® at each antenatal visit. The usefulness of the method was analysed by comparing the number of malaria episodes requiring treatment in the cohort with the number of prescriptions received by another group of pregnant women followed-up in routine antenatal care.

**Results:**

In the cohort group, the proportion of positive Paracheck_Pf_® episodes during antenatal clinics visits was 13.8%, while episodes of antimalarial prescriptions in the group which was followed-up routinely by antenatal personnel was estimated at 26.3%. Hence, the relative risk of the cohort for being prescribed an antimalarial drug was 0.53. Therefore, the attributable fraction of presumptive treatment avoided by systematic screening with Paracheck_Pf_® was 47%.

**Conclusions:**

Use of a rapid diagnostic test is useful, affordable and easy for adequate treatment of malaria in pregnant women. More powerful studies of the usefulness of introducing the test into antenatal care are needed in all heath centres in the country and in other tropical areas.

## Background

Malaria is a major public health problem, especially during pregnancy. In 2008, malaria affected more than 2 billion people, nearly 40% of the world population. More than 50 million women live in malaria-endemic areas, of whom 20% become pregnant each year, and half of whom develop complications of pregnancy due to malaria [1,2]. Placental inter-villous sequestration of malaria parasites during pregnancy can cause growth retardation by reducing nutrient intake and causing hypoxia [3,4] and thus low birth weight [3-5]. Placental infection with *Plasmodium* is associated with a 50% risk for low birth weight in areas where malaria is endemic [6]. Clinical malaria increases the risks for abortion, premature delivery and maternal death [7-9].

Sulfadoxine-pyrimethamine (SP) is a promising means of preventing the adverse effects of malaria, as first suggested by studies conducted in East Africa [10]. Hence, the World Health Organization (WHO) has recommended SP as a component of one of three packages to control malaria in pregnant women in Africa [11]: four antenatal visits, during which two doses of intermittent preventive (or presumptive) treatment with SP (IPTsp) spaced by at least 4 weeks are given by directly observed treatment; use of insecticide-treated nets (ITNs) to reduce the number of infective bites; and immediate, adequate treatment of clinical malaria. IPTsp involves administration of curative doses to asymptomatic women (two doses for women with negative HIV status and three doses for those infected with HIV). Because SP can be embryotoxic, it should be administered only after the 16th week (second trimester) of pregnancy [12,13]. The efficacy of IPTsp plus ITNs in reducing the burden of malaria during pregnancy is well proven [14-16] and cost-effective [17-20].

Lack of compliance of health care personnel with the new strategies could hamper implementation of IPTsp in particular [11,21]. Certain aspects of health care strategies determine their acceptability in the field [22]. Thus, the long-standing practice of prescribing antimalarial drugs only on the basis of a clinical presumption of malaria could result in under-administration of IPTsp in antenatal health care services.

Bardaji et al. in Mozambique [23] found that 77.4% of women attending antenatal clinics had clinical signs or symptoms suggesting malaria, and 92.9% of these were prescribed antimalarial drugs without laboratory confirmation of *Plasmodium* infection. [24]. This practice is likely to be common in tropical areas of Africa. Our recent study in the Central African Republic showed that laboratory analysis is rare before antimalarial drugs are prescribed for pregnant women [25]. Although widespread presumptive prescription and use of antimalarial agents could indisputably lead to prompt clearance of existing peripheral and placental *Plasmodium* infection and decrease the risk for low birth weight [26], unnecessarily wide use of antimalarial drugs is avoidable if preventive methods (IPTsp and LLINs) are used. The increasing resistance of *P. falciparum* to SP [27,28] jeopardizes the ability of this drug combination to eliminate the parasite completely, especially when it is sequestered in the placenta, hence, the importance of systematic screening for malaria during pregnancy [29], and use of rapid diagnostic tests (RDTs) is a possible strategy [30,31].

In public health care practice in the Central African Republic, malaria is diagnosed by analysing thick and thin blood smears; RDTs have not yet been introduced. The aim of this study was to assess the efficacy of systematic screening for malaria with RDTs during antenatal health care in reducing over-prescription of antimalarial drugs to pregnant women. The number of episodes of malaria confirmed by a RDT in a prospective cohort of pregnant women receiving routine antenatal care was compared with the number of antimalarial prescriptions in a control group of pregnant women at the same clinics.

## Method

### Study setting and design

This study was conducted in the two main public maternity clinics of Bangui, Central African Republic, the Castors Health Centre and the Amitié Hospital, between October 2009 and October 2011. In Bangui, the climate is tropical, with rainfall peaks from April to November and temperatures ranging from 19 *°*C to 33 °C. The main malaria parasite is *Plasmodium falciparum,* and malaria transmission is endemic, with peaks during the rainy season, although no data are available on the intensity of malaria transmission. Malaria accounts for more than 40% of morbidity in the country (CAR Ministry of Health, unpublished data).

The objective of the National Malaria Programme is to reduce morbidity and mortality related to malaria in the general population, especially among children under 5 years and pregnant women, to reach a coverage rate of at least 80% with each WHO package [11]. This study consisted of strict application of the three components of the WHO package to a cohort of women during their pregnancy. Longitudinal data on *Plasmodium* infection were compared with data collected from a matched control group followed-up routinely by the antenatal services staff.

### Ethical approval

Because there is no ethical committee in the country, this project was reviewed and approved by an *ad hoc* scientific committee of the University of Bangui in charge of validating scientific study protocols. The scientific committee of the ‘Ecole Doctorale des Sciences de la Vie et de la Santé de l’Université de la Méditerranée, Aix-Marseille’ also approved the study protocol and its amendments.

### Sample size

We used 25% as a proxy for the number of malaria episodes during pregnancy [29], and 50% as a proxy for the number of women usually prescribed antimalarial treatment [25]. The estimated sample size with 80% power at the 5% significance level was therefore 60 in each group.

### Procedures

Antenatal services staff were informed about the study, and we collected data in the context of usual non-malaria antenatal health care, including enrolment. During each working day, a maximum of two pregnant women from among those presenting at the clinics were randomly included in the cohort. The women were of all parities with a gestational age less than 28 weeks, from whom written informed consent was obtained. Women who were temporary residents of Bangui, had had a prior dose of IPTsp, gave a history of sensitivity to SP, quinine or an artemisinin derivative, had an illness requiring hospital admission or declined to join the study were excluded.

At enrolment, all women were given an ITN (supplied by the National Malaria Control Programme), and a finger-prick blood sample was obtained for preparation of slides and for diagnosis of *Plasmodium* infection with the Paracheck_Pf_® RDT (Orchid Biomedical Systems, India). We recorded sociodemographic data (age, address, occupational status and educational level) and also gestational age, gravidity, parity and HIV serological status.

During follow-up, an IPTsp dose (1500 mg sulfadoxine and 75 mg pyrimethamine) was administered after 16 weeks of gestation and a second dose at least 1 month later. Smear slide analysis and the RDT were performed systematically during each scheduled antenatal visit or at any unscheduled visit for women who reported malaria-like symptoms. Women with any symptom or clinical sign suggesting malaria [23] and with a positive result in the RDT and/or on a blood smear were given quinine (24 mg/kg of body mass) for 7 days. Women who reported sensitivity to quinine were given artemether 300 mg (20 mg) and lumefantrine (120 mg) if the gestational age exceeded 16 weeks. Women with a positive result in the RDT or on a thick smear but with no malaria symptoms were given one IPTsp dose; a smear was made 8 days later to verify any persistent parasitaemia or at any time earlier if a woman presented with symptomatic malaria, when quinine or artemether-lumefantrine was administered. All women were prescribed daily ferrous (400 mg) and folic (5 mg) supplements. All the antimalarial drugs were supplied by the ‘Unité de Cession du Médicament’, a public wholesaler that imports generic drugs.

Each woman in our cohort was matched on gravidity (1 or ≥ 2) and age (± 5 years) to another women delivering at the same maternity clinic within 1 week and from whom written informed consent was also obtained. Other criteria for enrolment in the control group were: known last date of menstruation (or gestational age at delivery), at least one antenatal visit, known HIV serological status and sleeping under an ITN. The following data were collected: socio-demographic information (age, address, occupational status and educational level), gestational age, gravidity, parity, HIV serological status, number of antenatal visits and number of malaria treatment episodes (with or without laboratory confirmation) during pregnancy.

At each antenatal visit, the RDT and blood smears were performed for each woman in the cohort. At delivery, these tests were performed on both maternal peripheral blood and placental blood. The placental blood was collected from the maternal paracentric side of the placenta after incision, and thick blood films were prepared from a droplet collected by aspiration through a 21-gauge needle attached to a 5-ml syringe [32,33]. Newborns were weighed on a mechanical infant body scale.

### Laboratory analysis

The maternal and placental thick smears were air-dried, stained with 4% Giemsa and analysed under a light microscope (*×* 100 oil immersion) to detect asexual forms of *P. falciparum*. The parasite density in maternal peripheral blood was determined from the number of parasites per 200 leukocytes on the assumption of an average leukocyte count of 8000/μl of blood. For maternal blood films, a result was considered negative if no parasites were detected per 200 leukocytes; for placental blood films, a result was considered negative if no parasites were detected per 100 microscope fields. All the slides were read independently by two microscopists. In case of a discrepancy, a third reading was done.

The RDT was performed according to the manufacturer’s guidelines and stored at room temperature (26–32 °C). Briefly, blood samples from the finger-prick and placenta were blotted into the sample well of the test device with the loop provided in the kit. Then, six drops (almost 300 μl) of clearing buffer were dispersed into the indicated well. The result was read after exactly 15 min. It was considered negative if a pink band appeared only in the control window and positive if, in addition to the control band, a distinct pink band also appeared in the test window. If no band appeared, the test was considered invalid and was repeated with a new device.

### Data analysis

Data were double-entered into EpiInfo software version 3.5.3, and the database was checked and data entry errors corrected with the EpiInfo software ‘data compare’ utility for finding differences between two tables. Statistical analysis was conducted with Stata 11.2 and MedCalc v11.6.1.

Categorical variables were compared by either the *χ*^2^ test or Fisher exact test, and quantitative variables were compared by the Student *t* test (Mann–Whitney *U* test). The level of significance (*P*) was fixed at 0.05 for all statistical tests.

We used the attributable fraction calculation procedure [34] to estimate avoidance of antimalarial drug prescription for pregnant women systematically screened for malaria. Thus, the risk for exposure to antimalarial drugs was calculated for each group, and then we calculated the relative risk (RR) of the women in the cohort for being treated in relation to the control group, and used the 1–RR formula to calculate the preventive fraction.

## Results

### Participant flow

**Figure 1:**
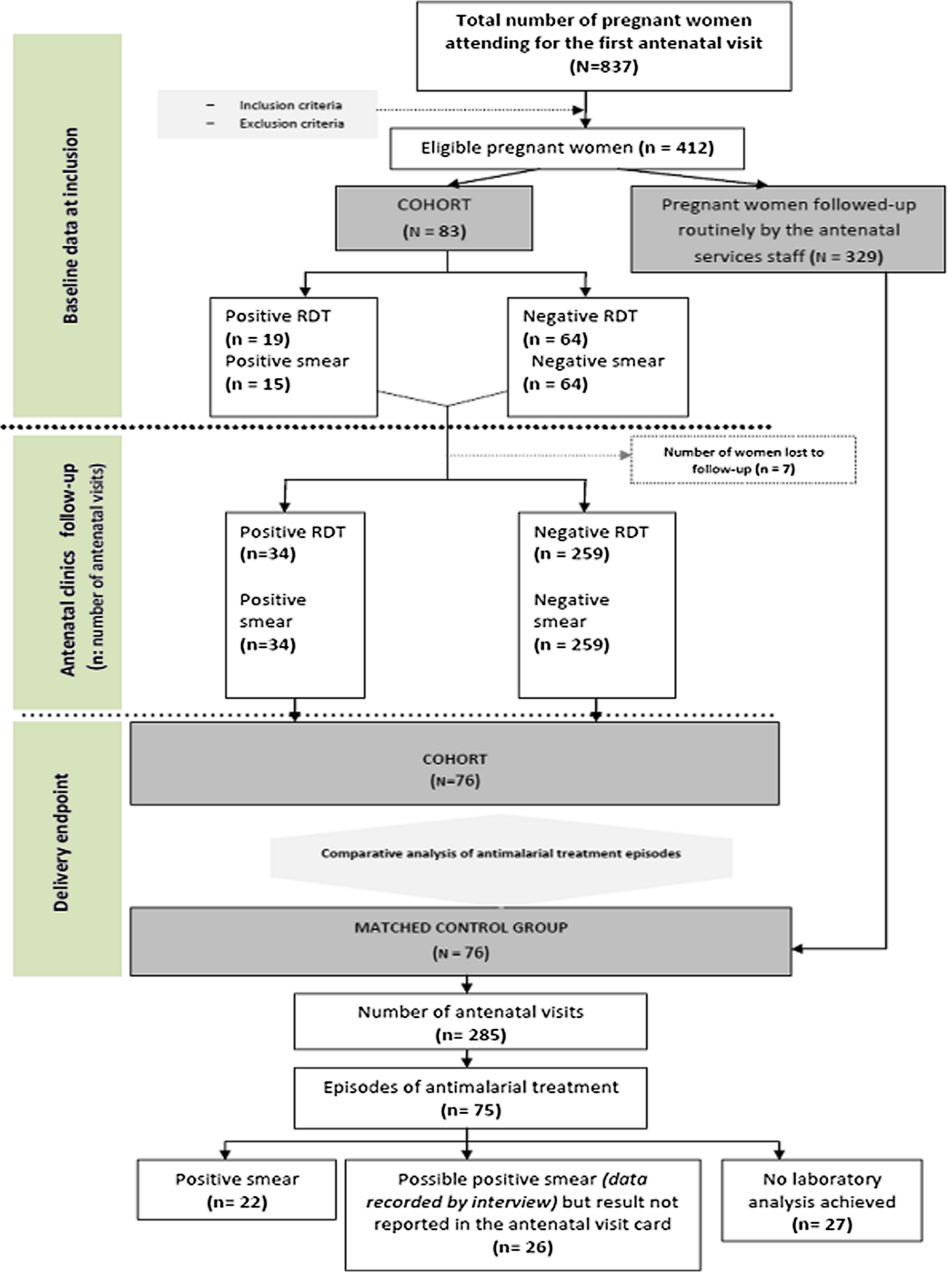
Study participant flow chart.

During the study period, 874 pregnant women presented at their first antenatal visit. At baseline, 412 women fulfilled the inclusion criteria. A cohort sample of 83 pregnant women was randomly recruited from among women who fulfilled the inclusion criteria. The remaining 329 women were followed-up routinely by the antenatal services staff, providing an eligible population from which the control group was recruited at delivery. In the cohort group, 76 women (91.6%) were followed-up until delivery. At this end-point, this cohort group was matched to the control group (Figure 1). Overall, the total number of antenatal visits was 369 in the cohort group and 285 in the control group.

### Characteristics of the study population

**Figure 2:**
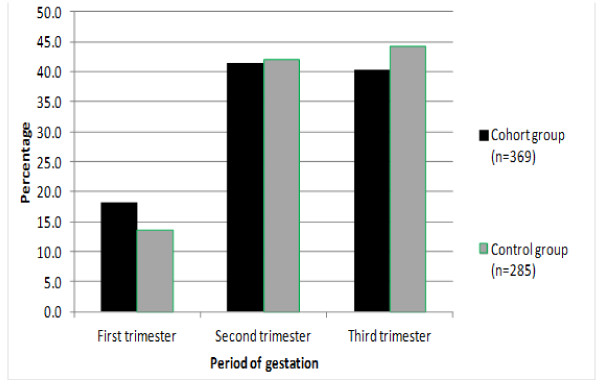
Numbers of antenatal visits according to gestational age in the cohort and control groups of pregnant women.

The average age of the women was 26 years (SD = 5 years), and the mean gravidity was 2 (range, 1–6), with no statistically significant differences between the groups. The serological prevalence of HIV infection was 7.6% (Table 1). The distribution of visits by length of gestation (in months) is shown in Figure 2. The IPTsp coverage rate with at least two doses was 93.4% in the cohort and 65.8% (50/76) in the control group. A statistically significant difference in the mean weight of birth of the infant was found between the two groups (P = 0.007).

**Table 1:** Characteristics of the study population

**Characteristic**	**Cohort (N = 76)**	**Control group (N = 76)**	**P value**
Mean age (years) (SD)	25.9 (5.4)	26.7 (3.8)	NS
Number of pregnancies			
0–1	32.4 (23)	29.6 (21)	NS
2	25.4 (18)	26.8 (19)	NS
≥ 3	49.3 (35)	50.7 (36)	NS
Educational level			
None	18.3(13)	14.1 (10)	NS
Primary school	33.8 (24)	38.0 (27)	NS
Secondary school	54.9 (39)	54.9 (39)	NS
HIV serological status	7.9 (6)	5.3 (4)	NS
Birth weight, mean g (SD)	3161 (351)	3297 (261)	0.007
Proportion of infants weighing < 2500 g	5.3	0.0	-

SD, standard deviation

### Results of diagnostic tests for malaria

In the cohort, 40 pregnant women (52.6%) presented clinical signs at baseline suggesting malaria. Of these women, 42.5% (17/40) had a positive RDT for malaria; none of the asymptomatic pregnant women had a positive result for malaria. During follow-up, 293 antenatal visits were made by the cohort group, and symptoms suggesting malaria were noted in 35.5% (104/293); however, positive RDT were found for only 29.8% (31/104). Overall, positive results for *P. falciparum* infection were found by the RDT at 13.8% of antenatal visits (51/369) and by thick blood smear analysis at 11.6% (43/369) of visits. Concordance was found for positive results in the RDT and in thick blood smears but not for negative results, as eight results were negative in the thick blood smear but positive with the RDT (15.7% discordance). Figure 1 shows a flow chart of these diagnostic tests.

Women positive results in the RDT were treated with either artemether-lumefantrine (45.1%, 23/51) or quinine (54.9%, 28/51). During follow-up, three asymptomatic malaria episodes were seen, with positive results in both the RDT and thick blood smear analysis. Each of these women received one dose of SP, and thick blood smears analysed 8 days later and at the following scheduled visit was negative.

In the control group, 75 prescriptions for a possible malaria episode were delivered by the antenatal services staff. Positive thick blood smears were documented on 35.0% of antenatal visit cards. These cards showed that 29.3% (22/75) prescriptions of antimalarial drugs were achieved after a documented positive laboratory result for malaria before treatment, while 36.0% (27/75) antimalarial drugs were achieved without laboratory analysis. For remaining prescriptions, laboratory analysis was not recorded in antenatal visit cards. However, interviewed women were reported that smear analysis were positive.

**Figure 3:**
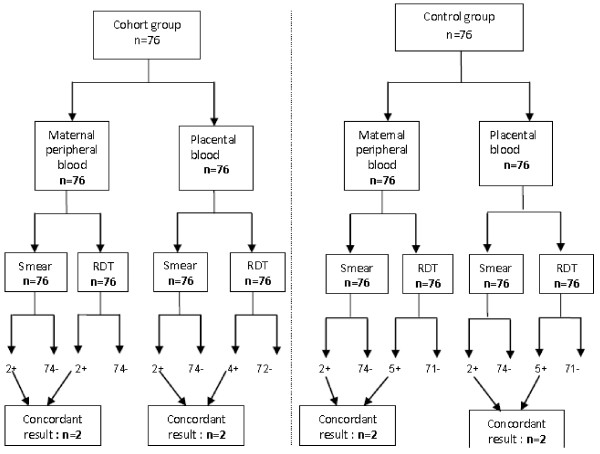
Results of thick blood smear analysis and Paracheck Pf® RDTs in the study and control groups of pregnant women at delivery.

At delivery, the results of the two tests were concordant for both the maternal peripheral blood smear and the placental blood samples in the two groups. Four placental blood samples from the cohort and only two from the control group were positive in the RDT. In the control group, five placental blood samples and two blood slides were positive with the RDT (Figure 3).

During this study, none of the tests was invalid.

### Effect of RDT results on malaria treatment

**Figure 4:**
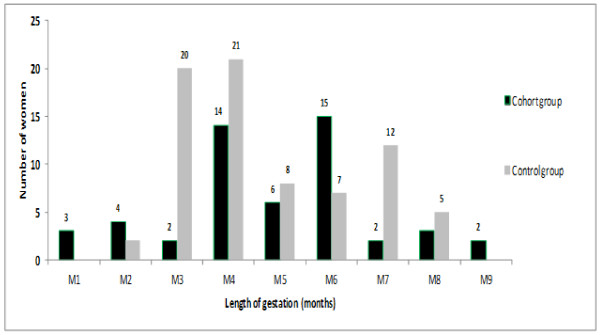
Distribution of malaria treatment episodes in the study and control groups of pregnant women.

Only 13.8% (51/369) of the pregnant women in our cohort needed antimalarial treatment after a positive RDT, while 26.3% (75/285) of the control group received such prescriptions. Antimalarial treatment was significantly more frequent in the control group than in the cohort (P = 0.0001). The relative risk of the cohort for being prescribed an antimalarial drug was 0.53; therefore, 47% of prescriptions were avoided with use of the RDT. The distribution of malaria treatment according to gestational age was, however, similar in the two groups (*U*-test *P* values > 0.05) (Figure 4).

## Discussion

Our findings highlight the value of using an RDT in managing malaria during pregnancy. The usefulness of RDTs in the diagnosis of malaria in patients presenting with symptoms or clinical signs is well established [35,36], although there has been concern that the number of malaria cases might be overestimated [37,38]. The performance of the ParacheckPf ® RDT in the diagnosis of malaria in pregnancy was recently appraised [39], and the use of RDTs in reducing the cost of treatment of malaria as compared with presumptive treatment has been demonstrated in children in Cameroon [40]. However, heat stability, vital to maintaining the sensitivity of this test in the field, is a concern [41,42], although there were no invalid tests.

The ParacheckPf ® RDT was more sensitive than microscopic analysis of thick blood smears, as found in other studies [38-40]. The priority is to determine whether an RDT can be positive when microscopy is negative [43]. The location of parasites in the deep blood circulation, particularly in the placenta, with release of antigens into the systemic circulation would explain a negative result in thick blood slides when an RDT is positive.

We observed a significant reduction in the number of antimalarial drug prescriptions in pregnant women when the ParacheckPf® RDT was used at each antenatal visit. This strategy therefore reduces unnecessary expenditure on drugs and also reduces unwarranted exposure of pregnant women to those drugs. Moreover, appropriate early treatment of malaria during pregnancy helps to eliminate parasites from both maternal blood and the placenta.

It has been estimated that malaria parasites are present in the placenta in only 20% of cases [44,45]. In our study, this proportion was 15.7%. As parasites sequestered in the placenta release antigens into the peripheral blood, they can be detected by RDT at any time of pregnancy.

One potential limitation of our study is the small sample, which precluded further analysis of birth weights; however, a study of three cohorts of a total of 3333 women conducted in Ghana [29] led to the same conclusion regarding the usefulness of screening with an RDT and treatment of positive cases. A second potential limitation was possible bias due to the “Hawthorne effect”, whereby people improve an aspect of their behaviour because they know they are being observed [46]. As our study was conducted in the office used by the staff of the antenatal clinics, they might have changed their attitude to improve the diagnosis of malaria by increasing their requests for laboratory analysis of thick blood smears.

## Conclusion

This study shows that routine screening of pregnant women for malaria can avoid unnecessary prescription of drugs. As any use of medical products during pregnancy should be avoided, the proportion of avoidable antimalarial drug exposure that we observed in this study could be important if it were extrapolated to other primary health care services.

Ensuring clearance of malaria parasites is essential for the control of malaria during pregnancy. Thus, the ease of use and relatively high sensitivity of the ParacheckPf® RDT in comparison with microscopy could allow prompt, radical treatment of malaria. Its integration into primary health care services should, however, be accompanied by regular supervision of the activities of the personnel involved. More studies are needed in other areas of the Central African Republic and other countries to assess the introduction of RDTs in malaria control in pregnant women.

## Competing interests

The authors declare that they have no competing interests.

## Authors’ contributions

AM, GS and JD conceived the study. AM organized and managed the study day-to-day and contributed to writing the manuscript. DD managed the RDT kits. ES, LSH and NM participated in field data collection. All authors contributed to the preparation of the manuscript and have approved the final version.

## Pre-publication history

The pre-publication history for this paper can be accessed here:

http://www.biomedcentral.com/1471-2458/12/482/prepub
